# Precision at the Bedside: Practical Efficacy of Clockwise Catheter Torque for Accurate Tip Positioning of Peripherally Inserted Central Catheters

**DOI:** 10.7759/cureus.50766

**Published:** 2023-12-19

**Authors:** Masayuki Akatsuka, Eriko Sugiyama

**Affiliations:** 1 Department of Intensive Care Medicine, Sapporo Medical University, School of Medicine, Sapporo, JPN; 2 Center for Graduate Medical Education, Sapporo Medical University Hospital, Sapporo, JPN

**Keywords:** resource-limited settings, clockwise catheter torque technique, ultrasound guidance, catheter malpositioning, peripherally inserted central catheters

## Abstract

Precise placement of peripherally inserted central catheters (PICCs) is essential for avoiding treatment risks and ensuring the success of treatment. This is typically performed under imaging guidance, but imaging modalities may not be accessible under resource-limited settings or alternative settings such as communicable disease isolation rooms. Here, we have proposed a new technique for the precise placement of PICCs through the application of clockwise torque. Application of the PICC with this technique in our patient resulted in the precise insertion of the catheter. As this method eliminates the need for imaging modalities, it has promise for application at the bedside and in resource-limited settings. Importantly, it presents a new aspect of catheterization protocols that could hold immense potential for the future. In the future, its efficiency needs to be verified in a larger number of patients under different settings and from different populations.

## Introduction

Peripherally inserted central catheters (PICCs) are used in clinical settings to give medications or parenteral nutrition. The precise positioning of PICCs remains a paramount concern in medical practice, given the potential ramifications of malpositioning, including inadvertent advancement into unintended vascular territories. Previous studies have underscored the significance of accurate PICC placement to mitigate the risks of malpositioning, and some of the commonly reported techniques for enhancing precision include ultrasound guidance and electrocardiography verification [1-3]. There is extensive literature on the safety of PICC placement, predominantly within the intensive care unit (ICU) setting [4,5]. However, questions persist regarding the feasibility of such procedures in environments characterized by limited resources and heightened procedural challenges, such as communicable disease isolation rooms. The present report tries to address this gap by unveiling a new technique involving clockwise catheter torque as a pragmatic solution for achieving accurate PICC tip positioning at the bedside. While existing methodologies primarily emphasize on imaging modalities, our approach involves a dynamic procedural maneuver that can be easily introduced to the established protocols and eliminates the need for imaging-based guidance. The lack of imaging modalities would make this procedure more feasible under resource-limited settings and would increase the possibility of precise PICC placement in these settings. Here, we present a case illustrating the successful application of the catheter torque technique to guide the PICC tip accurately at the bedside.

## Technical report

A 66-year-old man who was undergoing chemotherapy for follicular lymphoma was admitted to the ICU on account of the onset of pneumonia secondary to COVID-19 infection.

**Figure 1:**
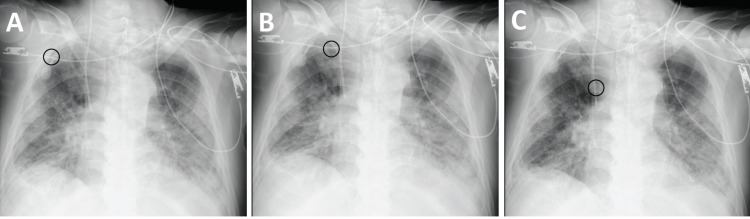
Chest radiograph showing the positions of the PICC tip (A) Placement of the tip in the subclavian vein. (B) Direction of the point of the tip toward the superior vena cava. (C) Placement of the tip in the lower half of the superior vena cava and the upper part of the right atrium. The images of (A) and (B) depict the application of torque while inserting the PICC. The black circle shows the positions of the PICC tip. PICC: peripherally inserted central catheter

On the 42nd ICU day, due to suspicion of catheter-related bloodstream infection, the central venous catheter in the right internal jugular vein needed to be replaced. We decided to place a PICC via the right ulnar cutaneous vein. We punctured the right ulnar vein with a needle under ultrasound guidance and inserted and placed a guidewire. A dilator was inserted along the guide wire, and the catheter was inserted and left in place. However, there was no hemovascular response after catheter placement, as assessed by evaluating blood reflux through the insertion of a syringe. Due to this lack of blood reflux, the clinician retracted the catheter into the subclavian vein and initiated a second attempt at insertion. During the second attempt, the catheter was carefully inserted with a clockwise rotation. Interestingly, this directional adjustment was successful, as evidenced by a conspicuous backflow of blood that was indicative of correct catheter tip placement (Figure 1). A confirmatory radiograph confirmed that the PICC had been successfully placed. The findings, in this case, highlight the potential efficacy of the clockwise catheter torque technique in guiding precise PICC tip placement at the bedside.

## Discussion

Two important clinical findings emerged from this case report. The first finding is that the PICC tip position can be adjusted at the bedside without the need for fluoroscopic guidance, thus improving the applicability of the procedure in settings with limited resources. The safety and efficacy of ultrasound-guided PICC placement have been demonstrated previously, but there are currently no studies on the safety of PICC insertion in limited environments where ultrasound guidance is not available [6]. Therefore, our technique represents an important shift in procedural considerations for PICC insertion and lays the ground for future work on PICC insertion in resource-limited settings.

The second important finding that emerged from this case was that the integration of torque during PICC insertion can enhance precision and ensure optimal placement of the catheter. Thus, the application of torque during PICC insertion is a potentially efficacious technique for guiding the catheter in the correct direction and affirms the application of torque maneuvers in vascular catheterization. This is consistent with the findings of previous literature [7]. However, some studies have suggested that fluoroscopic guidance is safer and more effective than echo guidance [5,8]. This difference may be explained by the nuanced nature of such techniques and differences in the contexts in which their efficacy was studied. In the context of our case, the observed benefits of torque present a promising avenue for refining PICC insertion protocols and advocate the inclusion of torque as a viable technique.

Our findings have a multifaceted impact on clinical practice. The demonstrated feasibility of safely guiding the PICC tip position at the bedside without fluoroscopy heralds a necessary shift in procedural adaptability that is pertinent in settings where fluoroscopic technology is inaccessible, as it offers clinicians a pragmatic alternative without compromising safety or precision. Furthermore, the potential efficacy of torque maneuvers during PICC insertion introduces a dynamic dimension to the procedural toolkit. Integrating torque as a technique holds promise for enhancing precision and addressing challenges posed by anatomical variations, ultimately fostering a more patient-centric approach.

There are several limitations of this case report that should be acknowledged to ensure a comprehensive understanding of its scope and applicability. First, the findings are based on a single case, which limits their generalizability. The uniqueness of individual patient characteristics, such as age, sex, medical history, physique, anatomical variations, and clinical conditions, means that the findings may not fully represent the broader population. A second limitation is that it is an operator-dependent technique. That is, the success of the clockwise catheter torque technique is dependent on the skill and experience of the practitioner, and the outcome could differ accordingly. Thus, the results may not be reproducible across different skill levels. Third, the lack of comparative data poses a serious limitation, as the report lacks comparative analysis with a control group or alternative techniques. Without such a comparison, it is challenging to ascertain whether the observed outcomes are specific to the clockwise catheter rotation technique or if they could be attributed to other factors. Fourth, as this is the first report on the technique, the findings need to be confirmed through further research. Future research with larger sample sizes, controlled trials, and comparative studies is required to validate the findings and establish the reliability and safety of the clockwise catheter rotation technique. Recognizing and addressing these limitations is essential for ensuring transparency, guiding future research directions, and providing a balanced interpretation of the study’s findings.

## Conclusions

This case report has profound clinical implications for bedside PICC guidance without fluoroscopy. The potential utility of torque maneuvers marks an important shift in procedural considerations. The findings will help clinicians navigate the complexities of PICC placement with adaptability and precision. This newfound understanding not only challenges established norms but also positions itself as a catalyst for innovation in catheterization protocols.
